# New eco-friendly animal bone meal catalysts for preparation of chalcones and aza-Michael adducts

**DOI:** 10.1186/1752-153X-6-60

**Published:** 2012-06-21

**Authors:** Yassine Riadi, Younes Abrouki, Rachid Mamouni, Mohammadine El Haddad, Sylvain Routier, Gérald Guillaumet, Saïd Lazar

**Affiliations:** 1 Laboratoire de Biochimie, Environnement & Agroalimentaire, URAC 36, Université Hassan II Mohammedia-Casablanca, Mohammedia, BP 146, 20650, Morocco; 2 Institut de Chimie Organique & Analytique, UMR CNRS 7311, Université d’Orléans, BP 6759, 45067, Orléans, Cedex 2, France; 3 Laboratoire de Chimie Organique, Université Ibn Zohr, BP 8106, 80000, Agadir, Morocco; 4 Equipe de Chimie Analytique & Environnement, Université Cadi Ayyad, BP 4162, 46000, Safi, Morocco

## Abstract

**Abstract:**

Two efficient reactions were successfully carried out using Animal Bone Meal (ABM) and potassium fluoride or sodium nitrate doped ABMs as new heterogeneous catalysts under very mild conditions. After preparation and characterization of the catalysts, we first report their use in a simple and convenient synthesis of various chalcones by Claisen–Schmidt condensation and then in an aza-Michael addition involving several synthesized chalcones with aromatic amines. All the reactions were carried out at room temperature in methanol; the chalcone synthesis was also achieved in water environment under microwave irradiation. Doping ABM enhances the rate and yield at each reaction. Catalytic activities are discussed and the ability to re-use the ABM is demonstrated.

**Results:**

For Claisen–Schmidt the use of ABM alone, yields never exceeded 17%. In each entry, KF/ABM and NaNO_3_/ABM (79-97%) gave higher yields than using ABM alone under thermic condition. Also the reaction proceeded under microwave irradiation in good yields (72-94% for KF/ABM and 81-97% for NaNO_3_/ABM) and high purity. For aza-Michael addition the use of ABM doped with KF or NaNO_3_ increased the catalytic activity remarkably. The very high yields could be noted (84-95% for KF/ABM and 81-94% for NaNO_3_/ABM).

**Conclusion:**

The present method is an efficient and selective procedure for the synthesis of chalcones an aza-Michael adducts. The ABM and doped ABMs are a new, inexpensive and attractive solid supports which can contribute to the development of catalytic processes and reduced environmental problems.

## Background

Heterogeneous catalytic reactions are interesting due to their well-documented advantages over homogeneous catalytic systems [1-3]. Animal Bone Meal (ABM) has emerged as an ideal basic heterogeneous catalyst as it is easily available in nature. The positive features of ABM also include high stability, ease of handling and regeneration, nontoxicity and the absence of other environmental hazards.

Our group has recently developed the preparation and use of Animal Bone Meal as a natural catalyst for C-S bond formation by thia-Michael addition [4], as a catalyst for crossed-aldol condensation [5], and as catalyst for synthesis of benzimidazoles, benzoxazoles, and benzothiazoles [6]. This new natural heterogeneous method led to *β*-sulfinyl adducts, α,α’-bis(substituted benzylidene)cyclo-alkanones, benzimidazoles, benzoxazoles, and benzothiazoles in very high yields after only a few minutes at room temperature.

As an extension of our research program concerning the development of this natural catalyst, we report in this paper two successive ABM catalyzed reactions (Scheme 1). In order to determine the best catalytic system, recycling assays, modifications of several parameters such as temperature, thermal or microwave irradiation and choice of solvents were investigated.

The first novel ABM catalyzed reaction led to chalcones 3 which constitute an important class of biologically active compounds [7-12]. They are commonly synthesized by Claisen-Schmidt condensation between acetophenones **1** and aromatic aldehydes **2** under acidic and basic homogeneous conditions or using basic heterogeneous catalysts.

Alumina [13], barium hydroxide [14-17], hydrotalcite [18], zeolite [19] natural phosphate either alone or activated with an ammonium salt [20] and hydroxyapatite derivatives [21-23] were previously used in heterogeneous reactions to facilitate separation and obtain pure products.

**Scheme 1** (a) Claisen-Schmidt condensation catalyzed by ABM, KF/ABM or NaNO_3_/ABM; (b) Aza-Michael addition by ABM, KF/ABM or NaNO_3_/ABM catalyst.

The second new ABM catalyzed reaction we tried is the Michael addition of an aromatic amine 4 on a chalcone 3 for the synthesis of *β*-amino carbonyl compounds 5. Previously, similar reactions have been carried out with no special activation (limited cases) [23,24] but most of them required a Lewis acid [25-27] and other stronger reaction conditions [28,29] to promote conjugation addition of amines to *α,β*-unsaturated carbonyl compounds. Solvent-free reactions, heterogeneous solid acids, ionic liquids, and high temperature have also been used to promote this reaction [30].

Unfortunately, as shown previously, many of these Claisen-Schmidt and aza-Michael procedures often required an excess of reagents, long reaction times, and used expensive heavy metal salts and drastic reaction conditions. The development of a simpler, eco-friendly and efficient catalytic method for the Claisen-Schmidt and aza-Michael reactions is therefore highly desirable. ABM and doped ABM could offer a good alternative in this respect.

### Catalyst preparation - characterization

ABM process preparation [31] offers the possibility to obtain a modified catalyst. KF/ABM and NaNO_3_/ABM doped bones were prepared by impregnating the natural product with an aqueous solution of potassium fluoride or sodium nitrate respectively. The mixture was stirred vigorously at room temperature, evaporated to dryness, dried and calcined at 800°C for 2 h. to obtain two novel active catalysts.

**Figure 1:**
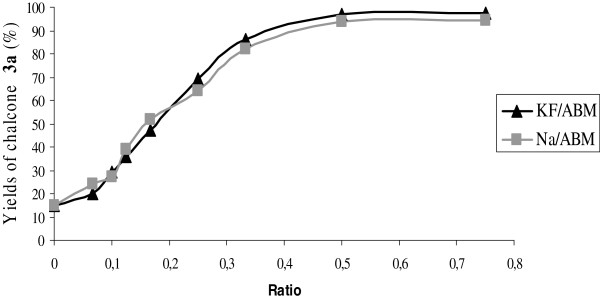
**Yields of 3a using KF/ABM and NaNO**_**3**_**/ABM with different weight ratios.**

In order to determine the best ratio r = m_0_ (KF or NaNO_3_)/m_1_ (ABM), we carried out the synthesis of chalcone 3a by condensation of benzaldehyde and acetophenone at room temperature in the presence of methanol (1 mL) and KF/ABM or NaNO_3_/ABM (50 mg) with weight ratios r = 3/4, 1/2, 1/3, 1/4, 1/6, 1/8, 1/10, 1/15 and 0 (ABM alone) respectively. In the presence of NaNO_3_ or KF alone, no product was observed under the reaction conditions; only the starting material was isolated (Figure 1).

**Figure 2:**
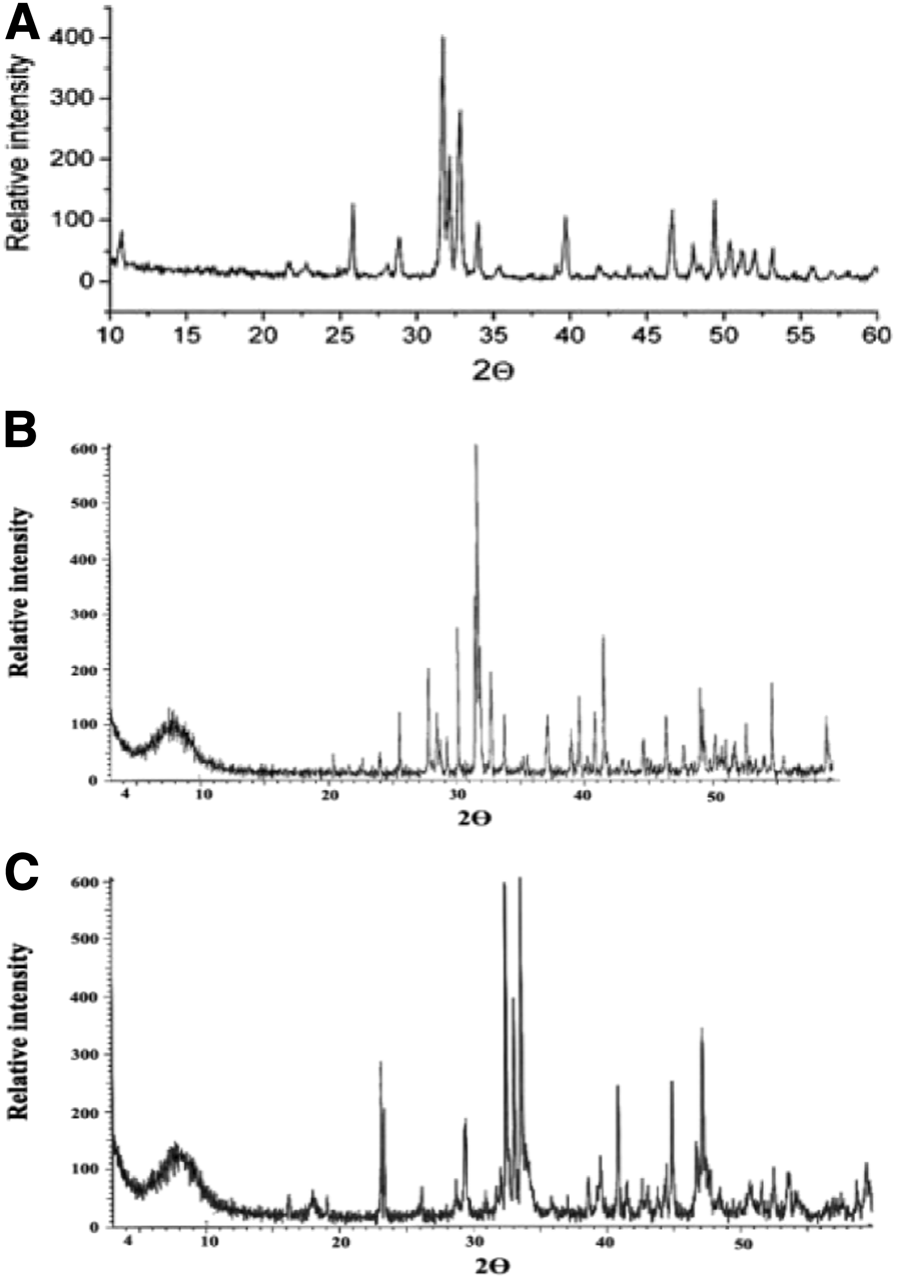
**(A) X-Ray diffraction patterns of ABM (B) X-Ray diffraction patterns of KF/ABM and (C) X-Ray diffraction patterns of NaNO**_**3**_**/ABM at weight ratio = 0.5.**

The conversion of the starting materials is maximal for a weight ratio KF or NaNO_3_/bones = 1/2. The new catalysts obtained under these conditions were characterized by X-ray diffraction (Figure 2).

We previously reported the elemental analysis of ABM which showed high levels of phosphate (56.3%) and calcium (36.8%), with a Ca/P ratio of 1.55. Only strong characteristic phosphate bands were observed in X-Ray spectra using doped ABMs. X-Ray diffraction analysis confirmed the presence of hydroxyapatite without solid material modifications.

The only remarkable fact is that the main modifications in the X-Ray diffraction patterns of KF/ABM and NaNO_3_/ABM are produced in the range of 2θ = 32–35°, in this range we have new lines with an increase in the intensity of other lines in that zone. These lines may correspond to sodium or potassium phosphate species.

### Optimizations for abms catalyzed Claisen-Schmidt condensations

Having thus demonstrated the ability to convert benzophenone and benzaldehyde into **3a** using the three newly prepared ABMs, we next envisioned optimizing the process of the reaction.

The reaction was therefore carried out using an equimolar amount of benzaldehyde and acetophenone (1.0 mmol) in various quantities of methanol. In the absence of solvent the reaction failed. The addition of methanol enhanced the yield up to 1.5-2.5 mL which appeared to be the best volume for achieving complete conversion of the starting material and obtaining **3a** in near quantitative yields. Efficient solvation of the reagents and contact optimizations between the active sites of the catalyst and the organic substrates could explain this result. Beyond 2 mL of MeOH, the dispersion phenomenon leads to a decrease in yields.

**Figure 3:**
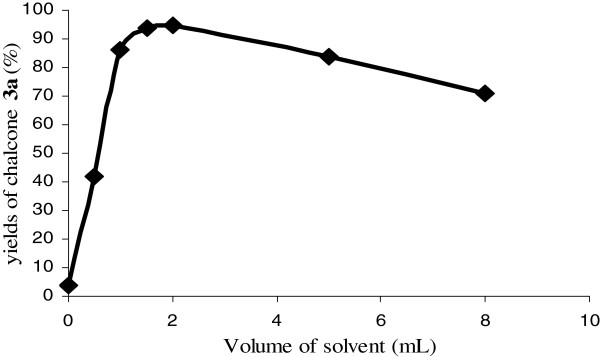
Volume of MeOH effect on chalcone 3a synthesis.

Various chalcones 3 were then prepared by reacting arylaldehydes 1 on ketones 2 in the presence of methanol as the solvent and catalyst (i.e. ABM or KF or NaNO_3_ doped ABMs) at room temperature (Figure 3). Under similar conditions in the absence of the catalysts, only the starting material was recovered, thus highlighting the role of this catalyst. At the end of the reaction a solvent addition, filtration and evaporation were realized in order to easily quantify the yield all the products were characterized using standard ^1^ H, ^13^ C NMR and IR spectrometry after re-crystallization.

**Table 1:** **Synthesis of chalcones 3 in presence of ABM, KF/ABM or NaNO**_**3**_**/ABM at room temperature in MeOH**

**Entry**		**Product**		**Yields**^**a**^** (%)/Time (h)**		
	**N° [ref]**	**X**	**Y**	**ABM**	**KF/ABM**	**NaNO**_**3**_**/ABM**
1	**3a**[32]	H	H	15/24	97/12	96/12
2	**3b**[33]	NO_2_	H	13/12	93/6	91/5
3	**3c**[32]	OCH_3_	H	5/12	94/9	91/6
4	**3d**[32]	Cl	H	10/12	90/6	93/6
5	**3e**[33]	CH_3_	H	5/12	91/9	86/9
6	**3f**[34]	F	H	17/12	90/6	92/6
7	**3g**[35]	H	NO_2_	6/12	97/6	95/6
8	**3h**[32]	OCH_3_	NO_2_	4/12	94/6	92/6
9	**3i**[35]	Cl	NO_2_	8/12	93/6	89/8
10	**3j**[36]	NO_2_	NO_2_	11/12	94/6	97/6
11	**3k**[37]	CH_3_	NO_2_	5/12	96/9	92/9
12	**3l**[38]	F	NO_2_	17/12	90/16	87/12
13	**3m**[32]	H	OCH_3_	9/12	85/12	88/12
14	**3n**[39]	NO_2_	OCH_3_	17/12	88/12	82/12
15	**3o**[32]	Cl	OCH_3_	2/12	89/12	91/12
16	**3p**[39]	CH_3_	OCH_3_	6/12	79/16	87/16
17	**3q**[32]	OCH_3_	OCH_3_	4/12	86/16	79/16
18	**3r**[40]	F	OCH_3_	14/12	92/12	94/12

^a^ Yields refer to isolated compounds.

Using ABM alone, yields never exceeded 17%. In each entry, KF/ABM and NaNO_3_/ABM gave higher yields than using ABM alone (Table 1). With our new catalysts, the reaction worked efficiently at room temperature in good yields, affording chalcones **3** in high purity. In the case of the synthesis of chalcones **3a**, **3l–3r**, the kinetic reactions were slowed down and required more time. The presence of an electron-donor group on acetophenones **2m-2r **(−OCH_3_) decreased their reactivity towards benzaldehydes whereas an electron-acceptor group such as -NO_2_ increased the reactivity of the corresponding acetophenones (**2g-2k**) except in case **2l**.

In general, it appeared that KF/ABM and NaNO_3_/ABM were more active than ABM, and that the addition of KF or NaNO_3_ to ABM also increased the activity of the heterogeneous catalysts.

Recovering and re-using the solid catalyst is one of the major interests of heterogeneous catalysis. For this purpose, the synthesis of **3a** was carried out using fresh and recovered catalyst NaNO_3_/ABM. The NaNO_3_/ABM was quantitatively recovered by simple filtration and regenerated by calcination at 400°C for two hours for each new reuse (usage).

**Table 2:** Studies on the reuse of NaNO_3_/ABM in the synthesis of chalcone 3a

**Entry**	**Round**	**Yield (%)**	**Recovered NaNO**_**3**_**/ABM (%)**
1	1	96	97
2	2	96	96
3	3	92	94
4	4	89	89
5	5	81	83

After the third recycling procedure, the yield of the reaction and the amount recovered NaNO_3_/ABM decreased slowly (Table 2). Nevertheless, the recycling procedure proved its efficiency and it should be noted that even in the fifth round of reuse of the catalyst the corresponding product **3a** was obtained in fairly good yield.

Even if chalcone synthesis proved its efficiency at room temperature, we next sought to find other conditions including replacing the organic solvent with water. For this new objective, a mixture of aldehydes **1**, acetophenones **2** (2.5 mmol) and doped ABMs was prepared in water and the suspension irradiated under microwave for the proper time at temperature 160°C and under pressure 10 bar. Simple purification led to the desired chalcones **3** (Table 3).

**Table 3:** **Synthesis of chalcones 3 in presence of KF/ABM or NaNO**_**3**_**/ABM under microwave irradiation**

**Entry**		** Product**		**Yields**^**a**^** (%)/Time (min)**	
	**N°**	**X**	**Y**	**KF/ABM**	**NaNO**_**3**_**/ABM**
1	**3a**	H	H	88/20	83/20
2	**3b**	NO_2_	H	82/20	84/20
3	**3c**	OCH_3_	H	82/30	89/20
4	**3d**	Cl	H	93/20	93/20
5	**3g**	H	NO_2_	91/20	96/20
6	**3h**	OCH_3_	NO_2_	85/20	81/20
7	**3i**	Cl	NO_2_	94/20	97/20
8	**3j**	NO_2_	NO_2_	91/25	93/20
9	**3m**	H	OCH_3_	79/30	82/20
10	**3n**	NO_2_	OCH_3_	84/30	84/30
11	**3o**	Cl	OCH_3_	78/30	86/30
12	**3q**	OCH_3_	OCH_3_	72/35	83/35

^a^ Yields refer to isolated compounds.

The reaction proceeded under microwave irradiation in good yields and high purity (Table 4). The reaction time was significantly reduced for each assay and products were obtained after only a few minutes. Electronic effects due to acetophenone and benzaldehyde substitutions persisted.

Compared the two methods the first reaction is performed at atmospheric pressure and the second one under pressure. In general, when the reaction was performed at atmospheric pressure the condensation has allowed the isolation of our products in good yields (Table 1). The reactions are relatively slow. The use of microwave decreases remarkably the reaction time and the attempted products were isolated in good yields after only few minutes (Table 2).

### Optimizations for ABMs catalyzed aza-Michael additions

The next step was to use our ABM catalysts for aza-Michael addition using some of the previously synthesized chalcones with amines **4**. For this new proposal, the catalyst (ABM, KF/ABM or NaNO_3_/ABM, 100 mg) was added to an equimolar mixture of aniline, *p*-methoxyaniline or benzylamine and chalcones (**3a-d**) in methanol. The mixture was stirred at room temperature until completion of the reaction (TLC). After filtration, the catalyst was washed with dichloromethane and after a simple purification step, the Michael adducts **5** were analyzed by ^1^ H, ^13^ C NMR and IR spectrometry. Reaction of 3a with aniline performed in MeOH without ABM was inefficient and the product **5a** was not detected.

In the presence of KF or NaNO_3_ alone, no 1,4-addition occurred, whatever the reaction time tried. In general the use of ABM alone led to the Michael adducts **5** in good (entry 1) to moderate yields (entry 12) and completion was obtained only after 12 h (except for the reaction of **3a** with aniline, 6 h).

**Table 4:** **Aza-Michael addition in presence of ABM, KF/ABM or NaNO**_**3**_**/ABM**

**Entry**		**Product**		**Yields**^**a**^** (%)/Time (h)**		
	**N° [ref]**	**R**	**X**	**ABM**	**KF/ABM**	**NaNO**_**3**_**/ABM**
1	**5a**[41]	C_6_H_5_	H	95/6	96/0.5	93/1
2	**5b**[42]	C_6_H_5_	NO_2_	86/12	93/1	94/1
3	**5c**[41]	C_6_H_5_	OCH_3_	75/12	93/3	91/3
4	**5d**[41]	C_6_H_5_	Cl	89/12	90/1	94/1
5	**5e**[43]	*p*-CH_3_OC_6_H_4_	H	86/12	97/2	94/1
6	**5f**[44]	*p*-CH_3_OC_6_H_4_	NO_2_	74/12	90/4	93/4
7	**5g**[42]	*p*-CH_3_OC_6_H_4_	OCH_3_	68/12	94/3	93/3
8	**5h**[45]	*p*-CH_3_OC_6_H_4_	Cl	78/12	92/3	91/2
9	**5i**[44]	C_6_H_5_CH_2_	H	59/12	95/12	91/12
10	**5j**[46]	C_6_H_5_CH_2_	NO_2_	67/12	92/6	93/6
11	**5k**[44]	C_6_H_5_CH_2_	OCH_3_	62/12	91/6	89/6
12	**5l**[44]	C_6_H_5_CH_2_	Cl	48/12	84/16	81/16

^a^ Yields refer to isolated compounds.

The use of ABM doped with KF or NaNO_3_ increased the catalytic activity remarkably. The very high yields (84-95% for KF/ABM and 81-94% for NaNO_3_/ABM could be noted. The rapidity of the reaction (0.5-4 h) using aniline and *p*-methoxyaniline proved the efficiency of these new catalytic systems. The sole limitations observed concern the use of benzylamine. In this case, the yields are higher but the reaction time increases to 6 h (entries 10, 11) and up to 16 h starting from the chlorochalcone **3d**.

Reuse of the doped ABM was investigated and a cycle of five aza-Michael additions was conducted. Each reaction produced the corresponding product **5a** in fairly good yield. KF/ABM was collected after each reaction and regenerated by calcination at 400°C for two hours for each new reuse (Table 5). During five cycles the catalyst was near quantitatively recovered but a slight decrease in yield was observed.

**Table 5:** Studies on the reuse of KF/ABM in the synthesis of product 5a

**Entry**	**Round**	**Yield (%)**	**Recovered KF/ABM (%)**
1	1	96	99
2	2	92	96
3	3	92	96
4	4	89	90
5	5	81	89

## Conclusions

ABM and KF or NaNO_3_ doped ABMs are efficient catalysts for performing Claisen-Schmidt condensation and aza-Michael additions under mild conditions. MeOH at room temperature and water under microwave irradiation offer two synthetic alternatives. These promising catalysts are very attractive and can be reused several times after simple filtration and calcination.

ABM and its KF or NaNO_3_ doped analogs are inexpensive and attractive solid supports which can contribute to the development of catalytic processes and reduce environmental problems.

### Experimental

General procedure for Claisen-Schmidt condensation: Chalcones 3 were prepared by reacting (2.5 mmol) arylaldehydes **1** and (2.5 mmol) ketones **2** in the presence of methanol (6.5 mL) as the solvent and 100 mg catalyst ABM or 50 mg of doped ABM at room temperature. CH_2_Cl_2_ (2 × 20 mL) was added, followed by simple filtration. The solution was concentrated and the crude material was purified by recrystallization.

General procedure for Claisen-Schmidt condensation under microwave irradiation: To a solution of aldehyde **1** (2.5 mmol) and acetophenone **2** (2.5 mmol) in water (300 μL) were added 100 mg of catalyst and the mixture was stirred at room temperature for 5 min. The mixture was irradiated by microwave for the appropriate time at 160°C. CH_2_Cl_2_ (2 × 20 mL) was added, followed by simple filtration. The solution was concentrated and the crude material was purified by recrystallization.

General procedure for the aza-Michael additions: To a flask containing an equimolar mixture (1.5 mmol) of amine **4** and chalcone derivative **3** in methanol (1.5 mL), 100 mg of catalyst (ABM, KF/ABM or NaNO_3_/ABM) was added and the mixture was stirred at room temperature until completion of the reaction (TLC monitoring). The reaction mixture was filtered and the catalyst washed with dichloromethane. After concentration of the filtrate under reduced pressure the residue was subjected to chromatography (*n*-hexane/EtOAc: 2/1) or recrystallization leading to the Michael adduct **5** as a solid.

**1,3-Bis-phenyl-propenone 3a**: mp 59-60°C [33]; ^1^ H NMR (CDCl_3_, 400 MHz): δ (ppm) 8.12 (2 H, d, *J* = 8 Hz), 7.63 (1 H, d, *J* = 16 Hz), 7.45 (1 H, d, *J* = 16 Hz), 7.46-7.64 (m, 6 H), 7.32 (2 H, t, *J* = 8.8 Hz) ppm; ^13^C NMR (CDCl_3_, 62.5 Hz): δ (ppm) 191.01, 145.30, 138.58, 135.25, 133.24, 131.00, 129.66, 129.31, 129.06, 128.93, 128.55, 122.44.

**1-(4-Nitrophenyl)-3-(4-Chlorophenyl)-propenone 3i**: mp 171-172°C [36]; ^1^ H NMR (CDCl_3_, 400 MHz): δ (ppm) 8.32 (2 H, d, *J* = 9.1 Hz), 8.11 (2 H, d, *J* = 8.8 Hz), 7.74 (1 H, d, *J* = 15.8 Hz), 7.59 (2 H, d, *J* = 8.8 Hz), 7.42 (1 H, d, *J* = 15.8 Hz), 7.36 (2 H, d, *J* = 8.8 Hz); ^13^ C NMR (CDCl_3_, 62.5 Hz): δ (ppm) 187.96, 150.64, 148.97, 142.01, 133.79, 133.19, 130.12, 129.84, 124.29, 121.26.

**1,3-Bis-(4-nitro-phenyl)-propenone 3j**: mp 192-194°C; ^1^ H NMR (CDCl_3_, 400 MHz): δ 8.06-7.81 (m, 8 H), 7.79 (d, 1 H, *J* = 16 Hz), 7.61 (d, 1 H, *J* = 16 Hz); ^13^C NMR (CDCl_3_, 62.5 Hz): δ 189.7, 142.0, 139.2, 137.6, 133.3, 132.6, 128.8, 128.7, 128.5, 125.1, 113.5.

**1-(4-Nitrophenyl)-3-(4-fluorophenyl)-propenone 3l**: mp 178-179°C; ^1^H NMR (CDCl_3_, 400 MHz): δ 8.38 (d, 2 H, *J* = 8 Hz), 8.31 (d, 2 H, *J* = 8 Hz), 8.18 (d, 2 H, *J* = 8 Hz), 7.88 (d, 1 H, *J* = 16 Hz), 7.82 (d, 2 H, *J* = 8 Hz), 7.60 (d, 1 H, *J* = 16 Hz); ^13^ C NMR (CDCl_3_, 62.5 Hz): δ 188.7, 144.0, 140.7, 131.1, 129.6, 128.3, 120.7, 113.7.

**1,3-Bis-(4-methoxyphenyl)-propenone 3q**: mp 103-104°C [33]; ^1^H NMR (CDCl_3_, 400 MHz): δ (ppm) 8.01 (d, 2 H), 7.77 (d, 1 H, *J* = 7.6 Hz), 7.59 (d, 2 H, *J* = 7.5 Hz), 7.41 (d, 1 H, *J* = 15.5 Hz), 6.95 (d, 2 H, *J* = 15.5 Hz), 6.90 (d, 2 H, *J* = 7.5 Hz), 3.83 (s, 3 H), 3.80 (s, 3 H). ^13^C NMR (CDCl_3_, 62.5 Hz): δ (ppm) 189.3, 164.4, 161.4, 143.9, 132.3, 131.6, 131.1, 128.7, 114.5, 113.3, 112.7, 55.7, 55.2.

**1,3-Diphenyl-3-phenylamino-propan-1-one 5a**: mp 170-171°C [42]; ^1^H NMR (CDCl_3_, 400 MHz): δ (ppm) 7.92 (d, 2 H, *J* = 8 Hz), 7.57 (t, 1 H, *J* = 8 Hz), 7.45 (t, 2 H, *J* = 8 Hz), 7.25 (t, 1 H, J = 8 Hz), 7.12–7.01 (m, 5 H), 6.79–6.77 (m, 3 H), 6.67 (t, 1 H, *J* = 8 Hz), 6.58 (d, 2 H, *J* = 8 Hz), 4.98 (dd, 1 H, J = 8; 5 Hz); 4.55 (br s, 1 H); 3.51 (dd, 1 H, *J* = 16; 5 Hz); 3.43 (dd, 1 H, *J* = 16; 8 Hz); ^13^C NMR (CDCl_3_, 62.5 Hz): 198.96, 197.80, 146.20, 136.48, 135.00, 133.61, 129.52, 128.79, 128.63, 128.18, 118.76, 114.00, 54.31, 40.92.

**3-(4-Methoxy-phenylamino)-1,3-diphenyl-propan-1-one 5e**: mp 165-166°C [45]; ^1^H NMR (CDCl_3_, 400 MHz): δ (ppm) 7.89 (d, 2 H, *J* = 8 Hz); 7.44 (d, 2 H, *J* = 8 Hz); 7.32 (t, 2 H, *J* = 8 Hz); 7.24 (t, 1 H, *J* = 8 Hz); 7.08 (d, 2 H, *J* = 8 Hz); 6.65 (t, 1 H, *J* = 8 Hz); 6.55 (d, 2 H, *J* = 8 Hz); 4.97 (dd, 1 H, *J* = 8; 4 Hz); 4.61 (br s, 1 H); 3.86 (s, 3 H); 3.45 (dd, 1 H, *J* = 16; 4 Hz); 3.34 (dd, 1 H, *J* = 16; 8 Hz); ^13^C NMR (CDCl_3_, 62.5 Hz): δ (ppm) 198.7, 158.5, 148.9, 136.7, 134.89, 133.3, 129.0, 128.6, 128.1, 117.7, 118.2, 114.4, 113.8, 55.2, 54.16, 46.3.

**3-Benzylamino-3-(4-methoxy-phenyl)-1-phenyl-propan-1-one 5k**: mp 100-101°C [46]; ^1^H NMR (CDCl_3_, 400 MHz): δ (ppm) 7.81 (2 H, *J* = 8 Hz), 7.51–7.12 (12 H, m), 4.5 (dd, 1 H, *J* = 8 Hz), 3.45-3.55 (m, 2 H), 3.2 (dd, 2 H, *J* = 16 Hz), 3.3 (s, 3 H), 3.21 (s, 1 H); ^13^C NMR (CDCl_3_, 62.5 Hz): 198.9, 144.8, 143.2, 140.4, 133.2, 132.8, 130.5, 129.0, 128.7, 128.6, 128.3, 128.1, 127.3, 127.1, 126.8, 122.1, 58.5, 51.6, 47.3, 46.5.

## Methods

Instrumentation and materials. ^1^H NMR and ^13^ C NMR were recorded on a Bruker Avance DPX250 spectrometer (400 MHz ^1^H, 62.89 MHz ^13^C) using tetramethylsilane as the internal standard, multiplicities were determined by the DEPT 135 equivalence, chemical shifts were reported in parts per million (ppm, *δ* units). Coupling constants were reported in units of hertz (Hz) if applicable.

Melting points were determined in open capillary tubes and are uncorrected. Flash chromatography was performed on silica gel 60 (40–63 mesh). Thin layer chromatography (TLC) was carried out on Merck silica gel 60 F_254_ precoated plates. Visualization was made with ultraviolet light. Chemicals products were obtained from the following sources: Aldrich and Acros organics.

Microwave (Biotage Initiator 2.5), Biotage microwave vials 2–5 mL.

## Competing interests

The authors declare that they have no competing interests.

## Authors' contributions

YR, SL and GG participated in study design and coordination, manuscript preparation and carriedout he synthetic experiments, SR participated in study design and coordination and manuscript reparation. All authors read and approved the final manuscript.
